# A new method applied for explaining the landing patterns: Interpretability analysis of machine learning

**DOI:** 10.1016/j.heliyon.2024.e26052

**Published:** 2024-02-09

**Authors:** Datao Xu, Huiyu Zhou, Wenjing Quan, Ukadike Chris Ugbolue, Fekete Gusztav, Yaodong Gu

**Affiliations:** a Research Academy of Medicine Combining Sports, Ningbo No. 2 Hospital, Ningbo, China; b Faculty of Sports Science, Ningbo University, Ningbo, China; c Faculty of Engineering, University of Pannonia, Veszprém, Hungary; d School of Health and Life Sciences, University of the West of Scotland, Scotland, United Kingdom; e Vehicle Industry Research Center, Széchenyi István University, Gyor, Hungary; f Department of Radiology, Ningbo No. 2 Hospital, Ningbo, China

**Keywords:** Landing pattern recognition, Clinical diagnosis, Biomechanics, Explainable machine learning, Layer-wise relevance propagation

## Abstract

As one of many fundamental sports techniques, the landing maneuver is also frequently used in clinical injury screening and diagnosis. However, the landing patterns are different under different constraints, which will cause great difficulties for clinical experts in clinical diagnosis. Machine learning (ML) have been very successful in solving a variety of clinical diagnosis tasks, but they all have the disadvantage of being black boxes and rarely provide and explain useful information about the reasons for making a particular decision. The current work validates the feasibility of applying an explainable ML (XML) model constructed by Layer-wise Relevance Propagation (LRP) for landing pattern recognition in clinical biomechanics. This study collected 560 groups landing data. By incorporating these landing data into the XML model as input signals, the prediction results were interpreted based on the relevance score (RS) derived from LRP. The interpretation obtained from XML was evaluated comprehensively from the statistical perspective based on Statistical Parametric Mapping (SPM) and Effect Size. The RS has excellent statistical characteristics in the interpretation of landing patterns between classes, and also conforms to the clinical characteristics of landing pattern recognition. The current work highlights the applicability of XML methods that can not only satisfy the traditional decision problem between classes, but also largely solve the lack of transparency in landing pattern recognition. We provide a feasible framework for realizing interpretability of ML decision results in landing analysis, providing a methodological reference and solid foundation for future clinical diagnosis and biomechanical analysis.

## 1 Introduction

Landing is one of many fundamental sports techniques, that can commonly be associated or accompanied by sports injuries, such as anterior cruciate ligament (ACL) injury, patellar tendinitis, ankle sprain, etc. [[1], [2], [3], [4], [5], [6], [7]]. Landing maneuvers are also frequently used in clinical injury screening, which include: 1) Assessment and screening of functional valgus collapse (an indicator of ACL injury) [8]; 2) Assessment of dynamic postural stability in ACL reconstruction where impaired sensorimotor control and mechanical stability can be identified among athletes [9]; 3) Screening for patients with chronic ankle instability [10], and so on. In clinical injury screening, clinical experts mainly performed quantitative description and analysis from the perspective of biomechanics, which includes biomechanical data such as kinematics (joint angle, spatiotemporal parameters, etc.), kinetics (ground reaction force, joint moment, etc.), and muscle status (muscle activation degree, muscle force, etc.). However, the quantitative descriptive analysis traditionally used in clinical screening is usually only for discrete variables at a specific time point, such as peak angle, peak force, peak moment, etc. Although this traditional approach has successfully solved many clinical injury screening problems, it also has the inherent limitation of losing a large amount of effective information when extracting low-dimensional single-time point discrete variables from high-dimensional time-continuous variables [11,12]. This is because in many cases it is not clear whether and to what extent a single pre-selected variable adequately represents the characteristics of that class of landing pattern. At the same time, the landing patterns are different in the situation of different injury conditions, different control/intervention conditions, and whether there is lower limb injury, which will cause great difficulties for clinical experts in clinical diagnosis. Therefore, a new method is urgently needed to explore the deeper characteristics of different landing patterns itself, to improve the effectiveness and accuracy of clinical injury screening.

Artificial intelligence (AI) and machine learning (ML) technologies are becoming more sophisticated as the economy and society develop, and their use in various industries to improve the efficiency of decision-making has also greatly reduced the burden on humans [13,14]. The healthcare industry is one of the many sectors that benefit from this, with AI and ML playing an important role in medical image-assisted diagnosis, pattern recognition of patient symptoms, identification of cancer tissue, etc [[15], [16], [17]]. This trend is also being recognized in the field of clinical gait analysis, where it has been successively used in gait pattern recognition and classification of patients with stroke [18], Parkinson's disease [19], cerebral palsy [20], osteoarthritis [21], and different functional gait disorders [22,23]. At the same time, advances in motion capture technology, mechanics sensing technology, and signal processing technology have made it possible to collect diverse and fine-grained human biomechanical data, providing the prerequisites for the application of big data-driven ML methods in the field of clinical biomechanics [12,23,24]. Although machine learning techniques have been very successful in solving a variety of clinical classification tasks and providing new insights from complex systems, they also suffer from an important limitation: black box effect [24,25]. That is, they all have the disadvantage of being black boxes and rarely provide and explain useful information about the reasons for making a particular decision [24,26]. This opaque operation and decision-making of most non-linear ML methods lead to a problem: the results of predictive recognition are hard to understand and uninterpretable, and this in turn undoubtedly affects the efficiency of ML applications in medical clinical diagnosis [27,28].

Based on this, the demand for ML methods for the interpretability of decision results in the field of clinical diagnosis continues to grow, and explainable machine learning (XML) has received increasing attention in recent years [28,29]. XML mainly refers to a series of machine learning methods that help the decisions and behaviors of artificial intelligence technology to be understood by humans, and which aims to illustrate how non-linear ML models work and the reasons for such predictive results [29]. The relevant application of XML in clinical diagnosis has increased the trust of experts in this field in the traceability of ML methods, and has been accepted by more and more researchers [30,31]. In recent years, an XML based on Layer-wise Relevance Propagation (LRP) has been proposed to address the problem of lack of interpretability of ML prediction results, which mainly measures the contribution and relevance of each input variable to the overall prediction results through backward propagation [26,32]. With its unique advantages in interpreting both linear and non-linear ML models, this method has been successfully applied to classification and recognition tasks in different scenarios, including clinical gait analysis, and has also been validated for its good interpretability performance in clinical diagnosis tasks [24,27,[33], [34], [35], [36], [37], [38]]. Whether XML can bring new challenges and opportunities for the application of landing pattern recognition in clinical diagnosis is not yet known. In other words, it is debatable whether XML can lead to new insights beyond the traditional “yes” or “no” simple discriminatory results.

Therefore, this work aims to investigate whether XML can help with landing pattern recognition and to what extent it can aid in the interpretation of prediction results. This work firstly compared the classification recognition performances of several classical classification models on two class landing tasks, and then constructed the XML model based on the neural network model with the best recognition performance combined with LRP to explain the model classification recognition results. At the same time, considering that biomedical signals are more abstract compared with signals such as image text, the evaluation of interpretability results is full of challenges [27,36,39]. To solve these challenges, the current study proposed two approaches to assess the computed interpretability results: 1) assessment from a statistical perspective; and 2) assessment from a clinical perspective. For statistical evaluation, a Statistical Parametric Mapping (SPM) technology [40] based on random field theory was used to detect statistical differences in the input signals, and then to verify whether interpretability results are reasonable based on statistical differences. For clinical evaluation, interpretable results were analyzed clinically by experienced experts in the field to assess their compatibility with clinical features.

Finally, our work mainly addresses the following two research questions: (1) which areas of input signals in the two class landing patterns are most relevant to the landing pattern recognition? (2) To what extent do these regions of input signals identified as most relevant for landing pattern recognition remain consistent with statistical evidence and clinical consensus assessment?

## 2 Materials and methods

**Fig. 1:**
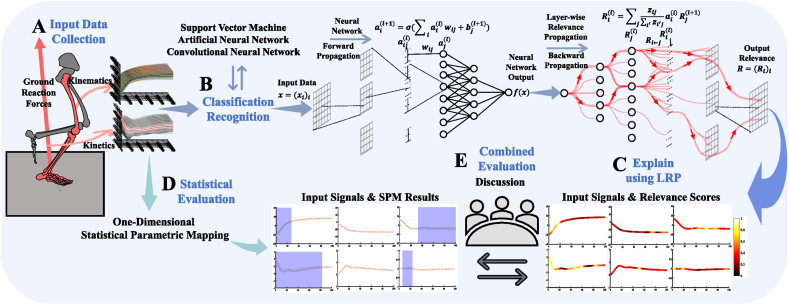
Overview of the proposed workflow for data collection, classification, and explanation in automated landing pattern recognition. Fig. 1A The single-leg landing movements of the subjects before and after the fatigue intervention were collected, and the three-dimensional kinematics and kinetics data of the landing leg during the landing phase were used as the input data of the model. Fig. 1B The three-dimensional kinematics and kinetics data as input signals to explore the recognizability of the two class landing patterns by three classical classification and recognition algorithm models and ZeroR classifier. Fig. 1C The ANN with the best performance in classification and recognition accuracy between classes was used as the forward propagation classifier to compute the input signals, and the output signals of ANN were used as the input of LRP to calculate the RS that can explain the difference of landing patterns through backward propagation. Fig. 1D The application of 1-SPM to evaluate the LRP results from a statistical perspective. Fig. 1E The results of these two aspects were evaluated and discussed together.

The flow diagram of the whole study procedure is shown in Fig. 1, which is mainly divided into three parts. This study selected two class landing datasets as the study input data, which were the single-leg landing between before fatigue intervention and after fatigue intervention (Fig. 1A). Firstly, three classical and widely used classification and recognition algorithm models (SVM: Support Vector Machine; ANN: Artificial Neural Network; CNN: Convolutional Neural Network) were selected for automated landing pattern classification tasks based on the three-dimensional kinematics and kinetics data of landing leg during the landing phase (Fig. 1B). At the same time, the ZeroR baseline was also computed by the ZeroR classifier for each classification task to verify the recognizability between classes. The classification task focuses on the recognizability of the differences between before fatigue and after fatigue landing patterns. Landing patterns represent the landing trends that subjects exhibit throughout the landing phase, which are usually specifically quantified by three-dimensional biomechanical data of the lower limbs.

Secondly, the ANN with the best performance in classification and recognition accuracy between classes was used as the forward propagation classifier, and the output of the ANN was used as the input of Layer-wise Relevance Propagation (LRP) to calculate the relevance score (RS) that can explain the difference of landing patterns through backward propagation (Fig. 1C). The relevance scores were used to determine the degree to which each joint contributed to the differences in recognizing landing patterns in each plane, that is, interpretable results on the recognizability of the differences between before fatigue and after fatigue landing patterns. After that, these results were evaluated from the statistical perspective (Fig. 1D) based on the approach of one-dimensional statistical parametric mapping (1-SPM). Finally, the results of these two aspects were evaluated and discussed together (Fig. 1E).

### 2.1 Landing Pattern Data

In landing movements, a large number of studies have shown that muscle fatigue will increase the risk of lower limb musculoskeletal injury during landing [[41], [42], [43], [44]]. In particular, the risk of lower extremity injury is generally increased in single-leg landings and can be detected from more pronounced changes in biomechanical data [2,41,[44], [45], [46]]. In other words, single-leg landing may be more difficult to maintain a safe state of the lower extremity than double-leg landing, thereby more effectively identifying risky biomechanical patterns [8]. Thus, a highly recognizable difference in landing pattern can be expected for single-leg landing between before and after fatigue intervention. At the same time, predictive explainability analysis can also be carried out on the formation of injury risk and the results of injury factors based on the differences in this landing pattern. Therefore, this study collected the single-leg landing movements of the subjects before and after the fatigue intervention, and the three-dimensional kinematics and kinetics data of the landing leg during the landing phase were used as the input signals of the algorithms model. A total of 56 healthy male subjects (age: 22.56 ± 5.13 years; body mass: 82.62 ± 13.38 kg; height: 1.85 ± 0.11 m) were recruited for this work to collect landing data before and after fatigue.

This study complies with the principles laid down in the Declaration of Helsinki. Ningbo University's Ethics Committee has accepted the study protocol (Approval Number: RAGH20210120), and all subjects supplied and signed written informed permission. Further details about landing data acquisition are provided in Supplementary Material Text 1. Finally, the data matrices of landing pattern were obtained as follow.

(1) $M_{before\;fatigue}=280\left(56_{subjects}\times 5_{trials}\right)\times 202\left(101_{kinematics}+101_{kinetics}\;\right)\times 9\left(3_{joint}\times 3_{plane}\right)$;
(2) $M_{after\;fatigue}=280\left(56_{subjects}\times 5_{trials}\right)\times 202\left(101_{kinematics}+101_{kinetics}\;\right)\times 9\left(3_{joint}\times 3_{plane}\right)$

### 2.2 Data classification

#### 2.2.1 Classification tasks

The main purpose of automated landing pattern classification is to detect high and low recognizability between different classes. To identify the single weights of each joint, each plane, kinematics, and kinetics in landing pattern recognition, the current study divides the input signal of the classification task into 9 algorithmic situations.

1) Both kinematics and kinetics signals: ${M1}_{before\;fatigue}=280\left(56_{subjects}\times 5_{trials}\right)\times 202\left(101_{kinematics}+101_{kinetics}\;\right)\times 9\left(3_{joint}\times 3_{plane}\right)$, ${\;M1}_{after\;fatigue}=280\left(56_{subjects}\times 5_{trials}\right)\times 202\left(101_{kinematics}+101_{kinetics}\;\right)\times 9\left(3_{joint}\times 3_{plane}\right)$;
2) Only kinematics signal: ${M2}_{before\;fatigue}=280\left(56_{subjects}\times 5_{trials}\right)\times 101_{kinematics}\times 9\left(3_{joint}\times 3_{plane}\right)$, ${M2}_{after\;fatigue}=280\left(56_{subjects}\times 5_{trials}\right)\times 101_{kinematics}\times 9\left(3_{joint}\times 3_{plane}\right)$;
3) Only kinetics signal: ${M3}_{before\;fatigue}=280\left(56_{subjects}\times 5_{trials}\right)\times 101_{kinetics}\times 9\left(3_{joint}\times 3_{plane}\right)$, ${M3}_{after\;fatigue}=280\left(56_{subjects}\times 5_{trials}\right)\times 101_{kinetics}\times 9\left(3_{joint}\times 3_{plane}\right)$;
4) Only ankle joint signal: ${M4}_{before\;fatigue}=280\left(56_{subjects}\times 5_{trials}\right)\times 202\left(101_{kinematics}+101_{kinetics}\;\right)\times 1_{ankle\;joint}\times 3_{plane}$, ${M4}_{after\;fatigue}=280\left(56_{subjects}\times 5_{trials}\right)\times 202\left(101_{kinematics}+101_{kinetics}\;\right)\times 1_{ankle\;joint}\times 3_{plane}$;
5) Only knee joint signal: ${M5}_{before\;fatigue}=280\left(56_{subjects}\times 5_{trials}\right)\times 202\left(101_{kinematics}+101_{kinetics}\;\right)\times 1_{knee\;joint}\times 3_{plane}$, ${M5}_{after\;fatigue}=280\left(56_{subjects}\times 5_{trials}\right)\times 202\left(101_{kinematics}+101_{kinetics}\;\right)\times 1_{knee\;joint}\times 3_{plane}$;
6) Only hip joint signal: ${M6}_{before\;fatigue}=280\left(56_{subjects}\times 5_{trials}\right)\times 202\left(101_{kinematics}+101_{kinetics}\;\right)\times 1_{hip\;joint}\times 3_{plane}$, ${M6}_{after\;fatigue}=280\left(56_{subjects}\times 5_{trials}\right)\times 202\left(101_{kinematics}+101_{kinetics}\;\right)\times 1_{hip\;joint}\times 3_{plane}$.
7) Only ankle joint signal: ${M7}_{before\;fatigue}=280\left(56_{subjects}\times 5_{trials}\right)\times 202\left(101_{kinematics}+101_{kinetics}\;\right)\times 1_{Sagittal\;plane\;}\times 3_{joint}$, ${M7}_{after\;fatigue}=280\left(56_{subjects}\times 5_{trials}\right)\times 202\left(101_{kinematics}+101_{kinetics}\;\right)\times 1_{Sagittal\;plane}\times 3_{joint}$;
8) Only ankle joint signal: ${M8}_{before\;fatigue}=280\left(56_{subjects}\times 5_{trials}\right)\times 202\left(101_{kinematics}+101_{kinetics}\;\right)\times 1_{Frontal\;plane\;}\times 3_{joint}$, ${M8}_{after\;fatigue}=280\left(56_{subjects}\times 5_{trials}\right)\times 202\left(101_{kinematics}+101_{kinetics}\;\right)\times 1_{Frontal\;plane}\times 3_{joint}$;
9) Only ankle joint signal: ${M9}_{before\;fatigue}=280\left(56_{subjects}\times 5_{trials}\right)\times 202\left(101_{kinematics}+101_{kinetics}\;\right)\times 1_{Transversal\;plane\;}\times 3_{joint}$, ${M9}_{after\;fatigue}=280\left(56_{subjects}\times 5_{trials}\right)\times 202\left(101_{kinematics}+101_{kinetics}\;\right)\times 1_{Transversal\;plane}\times 3_{joint}$;

At the same time, to ensure that the results of the LRP interpretation method employed after classification can be directly mapped to the original signal, the input signal was directly inputted into the classification model in this study. This was also done to better interpret the LRP results, thus not using techniques such as principal component analysis (PCA), which is commonly used in feature extraction and automatic pattern classification [12,33,47,48].

#### 2.2.2 Classification methods

For the nine input signals of the classification task, a total of three classical and widely used classification and recognition algorithm models were used in this study (SVM, ANN, CNN), which aims to give a more complete view of the problem under investigation and to make it possible to distinguish between task-specific and generic observations. The sample datasets of each task were randomly distributed uniformly to avoid errors during model training. Meanwhile, the data from the five successful trials for each subject were placed in the same subset during model training to ensure that the model performance was not affected by the same subject's data in different subsets. The eight-fold cross-validation was used in all classification algorithms. The data were randomly divided into eight parts, and then six of them were selected as the training set, one of them was selected as the validation set, and the remaining parts were used as the test set, repeating a total of eight times [49,50]. The final accuracy results obtained are based on the results generated from these eight training sessions, which greatly increases the accuracy of the results [49,50]. All the classification algorithm implementation were by self-written scripts based on the built-in function packages in MATLAB R2022a. Further details about description of classification methods are provided in Supplementary Material Text 2.

#### 2.2.3 Performance evaluation

The dataset of each class is randomly distributed uniformly to avoid errors during model training. Meanwhile, the data from the five successful trials for each subject were placed in the same subset during model training to ensure that the model performance was not affected by the same subject's data in different subsets. The eight-fold cross-validation was used in all classification algorithms. The data were randomly divided into eight parts, and then six of them were selected as the training set, one of them was selected as the validation set, and the remaining parts were used as the test set, repeating a total of eight times [49,50]. The final accuracy results obtained are based on the results generated from these eight training sessions, which greatly increases the accuracy of the results [49,50]. For each classification task, this study also computed the Zero-R baseline (ZRB). ZRB is the theoretical accuracy resulting from assigning class labels based on the prior probability of the class. For ZRB, the targeted label is always set to the class with the largest base in the training dataset [27].

### 2.3 Prediction explanation

In this study, only the LRP analyzed with the ANN algorithm model is provided since it consistently outperformed SVM and CNN algorithm model in terms of classification performance, and because its computational efficiency is relatively high. The complexity of neural networks comes from the interconnection of a large number of basic units, and its output is obtained by feedforward evaluation of these neurons. As shown on the right side of Fig. 1C, the RS that can explain the pattern recognition results is calculated by backward propagation. Based on the local redistribution rule, the Eq.1 can be obtained:

(1) $$R_{i}^{\left(l\right)}=\sum_{j}\frac{z_{ij}}{\sum_{i^{\prime }}z_{i^{\prime }j}}a_{i}^{\left(l\right)}R_{j}^{\left(l+1\right)}$$

and the $z_{ij}=x_{i}^{\left(l\right)}w_{ij}^{\left(l,l+1\right)}$, where the i is a neuron at the layer l+1, the $\sum_{j}\ldots$ runs over all upper layer neurons that are connected to neuron i. In general, LRP can compute the contribution of each feature to the classification result for dataset x, and the degree of such contribution can be reliably assessed to a certain extent (Each input feature x(d) contributes to a particular prediction f(x), where d is the input data of x(d) function). The l-th layer is modeled as a vector ${z=\left(z_{d}^{l}\right)}_{d=1}^{V\left(l\right)}$ with dimensionality V(l). For each dimension $z_{d}^{\left(l+1\right)}$ of vector z at layer l+1, LRP has an RS $R_{d}^{\left(l+1\right)}$. Each dimension $z_{d}^{l}$ of vector z towards the next layer l of the input layer contains an RS $R_{d}^{\left(l\right)}$, the Eq.2 can be obtained:

(2) $$f\left(x\right)=\ldots =\sum_{d\in l+1}R_{d}^{\left(l+1\right)}=\sum_{d\in l}R_{d}^{\left(l\right)}=\ldots =\sum_{d}R_{d}^{\left(1\right)}$$

The message $R_{i\leftarrow j}^{\left(l,l+1\right)}$ between neuron i and j represents the inter-hierarchical relevance, and these messages can be delivered along with each connection. The output f(x) is then backward propagated from one neuron to the next. The sum of incoming messages defined the relevance of neurons, and the sum runs over the sinks at layer l+1 for a fixed neuron i at a layer l:

(3) $$R_{j}^{\left(l\right)}=\sum_{k:i\;is\;input\;for\;neuron\;j}R_{i\leftarrow j}^{\left(l,l+1\right)}$$

The next neuron's input is directed in the direction specified during classification, then the total is computed over the sources at layer l for a fixed neuron k at layer l+1. In general, it can be expressed as:

(4) $$R_{k}^{\left(l+1\right)}=\sum_{i:i\;is\;input\;for\;neuron\;k}R_{i\leftarrow k}^{\left(l,l+1\right)}$$

The relevance of the linear network $f\left(x\right)=\sum_{i}z_{ij}$ is $R_{j}=f\left(x\right)$, and the straightforward decomposition by $R_{i\leftarrow j}=z_{ij}$. Through two monotone increasing functions (rectification function and hyperbolic tangent function), the pre-activation function $z_{ij}$ gives a reasonable method for determining the relative contribution of each neuron's $x_{i}$ to $R_{j}$. The association decomposition is chosen based on the proportion of local and global pre-activation:

(5) $$R_{i\leftarrow j}^{\left(l,l+1\right)}=\frac{z_{ij}}{z_{j}}*R_{j}^{\left(l+1\right)}$$

According to the Eq.5, through summing the correlations of all neurons in the upper layer i (combined Eq.3 and Eq.4), the overall relevance of all neurons in the following layer j can be determined:

(6) $$R_{i}^{\left(l\right)}=\sum_{j}R_{i\leftarrow j}^{\left(l,l+1\right)}$$

Based on the Eq.6, the relevance propagates from the upper layer to the lower layer till it attains the input feature x(d), where the hierarchical eigen-decomposition necessary for the choice f(x) is provided by the relevance $R_{d}^{\left(1\right)}$. More information is available in the previous study [32,51]. Overall, LRP determined the RS between each variable and the model predicted results, and standardized the RS of LRP derived to their respective values for comparison. The average of all relevant modes was rectified by calculating the smoothing, where the previous and next points were weighted by 25% and the current point was weighted by 50% (total weight is 100%). The input landing pattern-related data was collected in the time domain, its adjacent values depend on each other, so applying the smoothing process can reduce the calculated RS fluctuation without affecting the general pattern [24]. The entire smoothing process was repeated three times (by Gaussian Filter) before scaling the smoothed correlation pattern to 0 (indicates the lowest correlation) - 1 (indicates the highest correlation). In this study, the algorithm implementation by self-written scripts was based on the LRP toolbox in MATLAB R2022a [52].

### 2.4 Statical evaluation

In this study, Statistical Parametric Mapping (SPM) was used to evaluate the LRP results (derived RS), which has recently been widely used in the field of landing and gait analysis [1,46,[53], [54], [55]]. SPM is a technique for conducting time series statistical analysis of continuous data collected over a period of time [40,56]. Throughout the full performance, it can test and examine statistical differences in data that change over time. A comprehensive and objective statistical result is obtained through statistical analysis of the oversimplified vector trajectory, which can factually instruct the investigation of complex biomechanical structures [56]. The primary benefits of SPM are the presentation of statistical data in the original sampling space and the absence of the parameterization procedure [40,56]. As SPM is entirely data-centric relative to interpretable machine learning algorithms, and the output results of SPM and LRP are both based on the same input signal space, it is entirely appropriate to use SPM as a model-independent method to assess the quality of LRP-derived results [27].

For the implementation of SPM, the open-source MATLAB script (paired-samples T-test) of One-Dimensional SPM (SPM 1D) was employed to test the statistical differences, and the significance threshold was set as 0.05 [40,56]. The output of the SPM provides t-values for each time point of the explored one-dimensional time series input signal, as well as the time series interval corresponding to the determined significance threshold. A t-value above the significance threshold indicates that the difference in the corresponding part of the time series is statistically significant (as shown in the blue shaded part corresponding to Fig. 4A, Fig. 5A, and Fig. 6A). In addition, the effect size was calculated by transforming the obtained t-values to the r of Pearson's correlation coefficient based on Rosenthal's study [57]. The effect size is independent of the significant size and is divided into three regions to provide an indicator to distinguish a given signal [58].

## 3 Results

### 3.1 Kinematics and kinetics data waveform of landing pattern

**Fig. 2:**
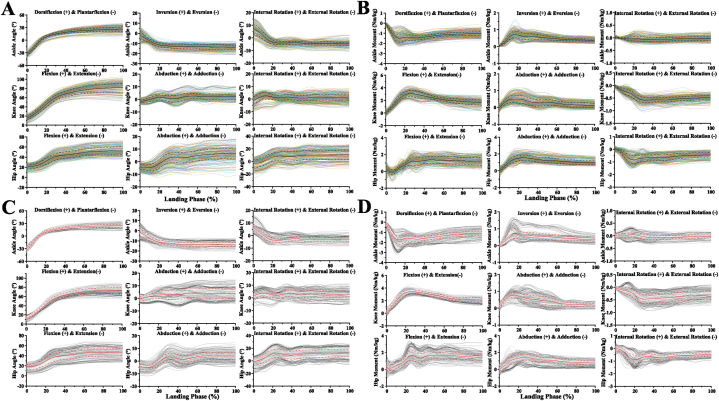
Visualization of joint angle and joint moment of each joint (ankle, knee, hip) of each plane (sagittal, frontal, transversal) during the landing phase between before fatigue and after fatigue single-leg landing. Fig. 2A The raw joint angle data waveform during the landing phase of the single-leg landing of before fatigue intervention. Fig. 2B The raw joint moment data waveform during the landing phase of the single-leg landing of before fatigue intervention. Fig. 2C The raw joint angle data waveform during the landing phase of the single-leg landing of after fatigue intervention. Fig. 2D The raw joint moment data waveform during the landing phase of the single-leg landing of after fatigue intervention. Y-axis means the 0%–100% landing phase. The anatomical definition is shown at the top of the figure. The color curve is the full test data set for the landing of before fatigue intervention, and the black curve and the line are the mean and standard deviation of these data sets. The gray curve is the full test data set for the landing of after fatigue intervention, and the red curve and the line are the mean and standard deviation of these data sets.

The raw kinematics (joint angle) and kinetics (joint moment) data waveform of each joint (ankle, knee, hip) of each plane (sagittal, frontal, transversal) during the landing phase between before fatigue and after fatigue single-leg landing are shown in Fig. 2. Fig. 2A and B display the raw joint angle and moment data waveform during the landing phase of the single-leg landing of before fatigue intervention, respectively. Fig. 2C and D show the raw joint angle and moment data waveform during the landing phase of the single-leg landing of after fatigue intervention, respectively.

### 3.2 Classification results

**Fig. 3:**
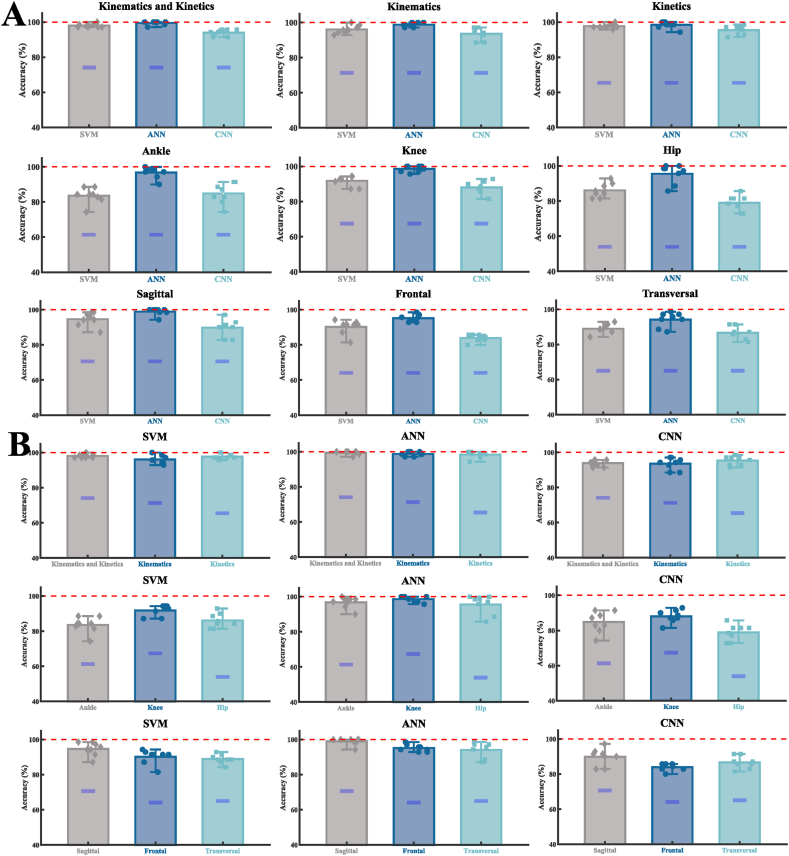
Detailed results of the prediction accuracy rate were obtained for the three different classification algorithm models (SVM, ANN, CNN). Fig. 3A Comparison results of classification accuracy of nine classification tasks under three algorithm models. Fig. 3B Comparison results of classification accuracy of three algorithm models under nine classification tasks. Bar graph with scatter points of the classification and recognition accuracy rate acquired through eight-fold cross-validation (a total of eight individual scatter points).

The result distribution of prediction accuracy rate and ZRB was shown in Fig. 3, and the details of means and standard deviations and ZRB were shown in Table 1. Comparison results of classification accuracy of nine classification tasks under three algorithm models were shown in Fig. 3A. The ANN model showed better classification performance than SVM and CNN in all nine classification tasks, especially in the three classification tasks that took separate ankle, knee, and hip data as input signals. Comparison results of classification accuracy of three algorithm models under nine classification tasks were shown in Fig. 3B. The classification performance that is based on both kinematics and kinetics as input signals are better than the classification performance that is based on only kinematics or only kinetics as input signals. The classification performance that is based on the knee data as input signals was better than the classification performance that is based on the ankle data or only hip data as input signals. The classification performance that is based on the sagittal plane data as input signals was better than the classification performance that is based on the frontal plane data or only transversal plane data as input signals. The prediction accuracy based on three classification algorithm models both were higher than the ZRB results.

**Fig. 4:**
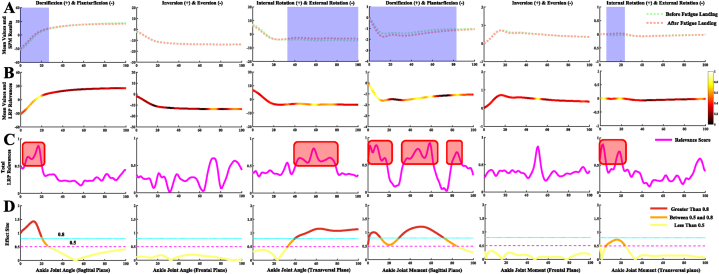
Detailed overview for the classification of the ankle joint kinematics and kinetics during the landing phase between before fatigue and after fatigue single-leg landing. Fig. 4A Comparison of the ankle joint kinematics and kinetics in the sagittal, frontal, and transversal plane between before fatigue and after fatigue single-leg landing. The anatomical definition is shown at the top of the picture. The blue shaded part indicates that there has a statistical difference in the landing phase in this section. Fig. 4B Mean values of all test trial datasets, color-coded by input RS for both classes acquired through LRP. The brighter colors mean high relevance variables, and the darker colors mean low relevance variables. The brighter the color, the greater the contribution to landing pattern recognition. Fig. 4C Detailed line plot of the RS for both classes acquired through LRP during the landing phase. The red shaded part represents the region where the RS obtained by LRP highly coincide with the SPM results. Fig. 4D Detailed line plot of the effect size for both classes during the landing phase. The situation description is shown at the bottom of the figure.

**Fig. 5:**
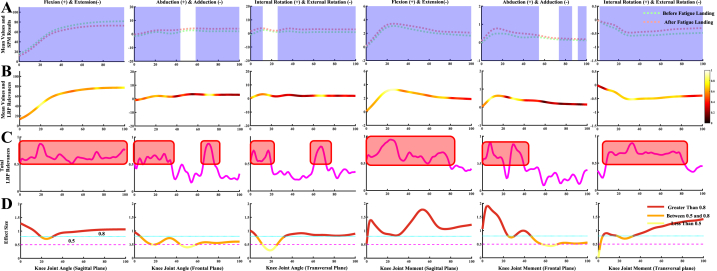
Detailed overview for the classification of the knee joint kinematics and kinetics during the landing phase between before fatigue and after fatigue single-leg landing. Fig. 5A Comparison of the knee joint kinematics and kinetics in the sagittal, frontal, and transversal plane between before fatigue and after fatigue single-leg landing. The anatomical definition is shown at the top of the picture. The blue shaded part indicates that there is a statistical difference in the landing phase in this section. Fig. 5B Mean values of all test trial datasets, color-coded by input RS for both classes acquired through LRP. The brighter colors indicate high relevance variables, and the darker colors indicate low relevance variables. The brighter the color, the greater the contribution to landing pattern recognition. Fig. 5C Detailed line plot of the RS for both classes acquired through LRP during the landing phase. The red shaded part represents the region where the RS obtained by LRP highly coincide with the SPM results. Fig. 5D Detailed line plot of the effect size for both classes during the landing phase. The situation description is shown at the bottom of the figure.

**Fig. 6:**
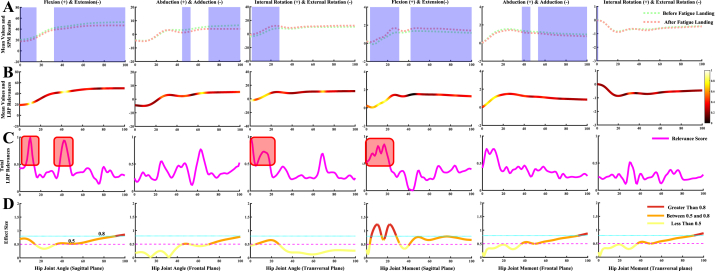
Detailed overview for the classification of the hip joint kinematics and kinetics during the landing phase between before fatigue and after fatigue single-leg landing. Fig. 6A Comparison of the hip joint kinematics and kinetics in the sagittal, frontal, and transversal plane between before fatigue and after fatigue single-leg landing. The anatomical definition is shown at the top of the picture. The blue shaded part indicates that there is a statistical difference in the landing phase of this section. Fig. 6B Mean values of all test trial datasets, color-coded by input RS for both classes acquired through LRP. The brighter colors reprent high relevance variables, and the darker colors reprent low relevance variables. The brighter the color, the greater the contribution to landing pattern recognition. Fig. 6C Detailed line plot of the RS for both classes acquired through LRP during the landing phase. The red shaded part represents the region where the RS obtained by LRP highly coincide with the SPM results. Fig. 6D Detailed line plot of the effect size for both classes during the landing phase. The situation description is shown at the bottom of the figure.

**Table 1:** Detailed values of the prediction accuracy rate were obtained for the three different classification algorithm models (SVM, ANN, CNN) and ZRB in nine classification tasks. Means and standard deviations were obtained based on eight-fold cross-validation.

Input Signals	SVM (%)	ANN (%)	CNN (%)	ZRB (%)
Kinematics and Kinetics	98.04 ± 1.06	99.46 ± 1.06	93.93 ± 1.83	74.11
Kinematics	96.07 ± 2.38	98.75 ± 1.19	93.57 ± 3.41	71.30
Kinetics	97.68 ± 1.31	98.39 ± 1.94	95.36 ± 2.62	65.41
Ankle	83.57 ± 4.52	96.79 ± 3.22	84.82 ± 5.96	61.33
Knee	91.79 ± 3.03	98.57 ± 1.71	88.04 ± 3.58	67.36
Hip	86.07 ± 4.09	95.54 ± 5.42	78.93 ± 4.50	53.93
Sagittal	94.64 ± 3.87	98.93 ± 1.98	89.82 ± 4.86	70.63
Frontal	90.18 ± 4.07	95.18 ± 2.01	83.93 ± 1.98	64.08
Transversal	88.93 ± 2.62	94.11 ± 4.14	86.61 ± 3.58	65.01

### 3.3 Explainability and statistical evaluation results

Detailed recognizability results and statistical evaluation of the differences between before fatigue and after fatigue single-leg landing patterns are shown in Fig. 4, Fig. 5, Fig. 6.

The classification and recognition results of the ankle joint kinematics and kinetics during the landing phase between two class landing patterns are shown in Fig. 4. Fig. 4A showed the comparison results of the two class landing patterns in ankle joint kinematics and kinetics, and SPM results show that the landing pattern differences are mainly concentrated in the sagittal and transversal plane (blue shaded part). Fig. 4B showed the mean values of all test trial datasets, and the results of color-coded input RS for both classes acquired through LRP. Fig. 4C shows the size of detailed RS during the landing phase, and the area with high RS in the ankle joint is consistent with the statistical results of SPM (red shaded part). Fig. 4D shows the effect size for both classes during the landing phase, where the portion above 0.5 (red and orange lines) is also basically consistent with the high RS area, especially those parts above 0.8 (red lines). In general, the RS derived from LRP in identifying the differences between the two class landing patterns generally agreed with the statistical results in the ankle joint.

The classification and recognition results of the knee joint kinematics and kinetics during the landing phase between two class landing patterns are shown in Fig. 5. Fig. 5A shows the comparison results of the two class landing patterns in knee joint kinematics and kinetics. The SPM results indicated that the landing pattern differences are observed at all three anatomical planes (blue shaded part). Fig. 5B shows the mean values of all test trial datasets, and the results of color-coded input RS for both classes acquired through LRP. Fig. 5C shows the size of detailed RS during the landing phase, and the area with high RS in the knee joint is consistent with the statistical results of SPM (red shaded part). Fig. 5D shows the effect size for both classes during the landing phase, where the portion above 0.5 (red and orange lines) is also consistent with the high RS area. In general, the RS derived from LRP in identifying the differences between the two class landing patterns also agreed with the statistical results in the knee joint.

The classification and recognition results of the hip joint kinematics and kinetics during the landing phase between two class landing patterns were shown in Fig. 6. Fig. 6A shows the comparison results of the two class landing patterns in hip joint kinematics and kinetics, and SPM results show that the landing pattern differences are mainly concentrated in the sagittal and frontal plane (blue shaded part). Fig. 6B shows the mean values of all test trial datasets, and the results of color-coded input RS for both classes acquired through LRP. Fig. 6C shows the size of detailed RS during the landing phase, and the partial area with high RS in the hip joint was consistent with the statistical results of SPM (red shaded part). The RS derived from LRP in the hip sagittal and frontal planes during the late landing phase is inconsistent with the statistical results of SPM, although there was a statistical difference a high RS at this stage was not producted. Fig. 6D shows the effect size for both classes during the landing phase, where the portion above 0.5 (red and orange lines) is partially consistent with the high RS area.

**Fig. 7:**
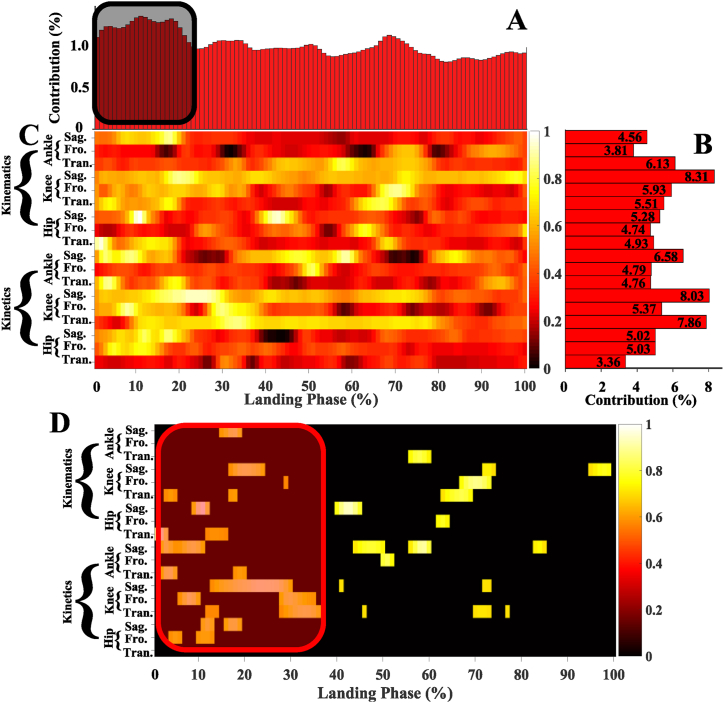
Detailed explanation results of landing pattern difference using LRP. Fig. 7A Relative contribution of variables during the overall landing phase. Fig. 7B Summed contribution of the relevance of each joint (ankle, knee, hip) of each plane (sagittal, frontal, transversal) of kinematics and kinetics trajectories. Fig. 7C Detailed distribution of RS during each joint of each plane of kinematics and kinetics. Fig. 7D Detailed distribution of the 169 highly relevant variables (RS greater than 0.7) during each joint of each plane of kinematics and kinetics. The brighter colors indicate high relevance variables, and the darker colors indicate low relevance variables. The model relied more on brighter color variables; the darker color variables had less relevance with correctly classified landing patterns.

Detailed results of RS derived from LRP for explaining the landing pattern difference between before fatigue and after fatigue single-leg landing were shown in Fig. 7. Fig. 7A shows the relative contribution of variables during the overall landing phase, the variables recorded at every 1% of the landing phase interval were related to successfully matching the landing pattern between before fatigue and after fatigue single-leg landing. The variable contribution during the 1%–22% landing phase reached 27.06%, which indicated that the contribution of the early landing phase to the recognition of landing patterns was greater (black shaded area). Fig. 7B shows the summed contribution of the RS of each joint of each plane of kinematics and kinetics trajectories. For each joint, the largest summed contribution rate of RS was 41.02% in the knee joint, followed by 30.62% in the ankle joint and 28.36% in the hip joint. For each plane, the largest summed contribution rate of RS was 37.78% in the sagittal plane, followed by 32.55% in the transversal plane and 29.67% in the frontal plane. The knee flexion–extension angle variable was the most relevant trajectory variable in landing pattern recognition, and the contribution rate of RS reached 8.31%. Secondly, the knee flexion–extension moment, knee internal–external rotation moment, ankle dorsiflexion-plantarflexion moment, ankle internal–external rotation angle, were the followed relevant trajectory variables in landing pattern recognition, and the contribution rate of RS reached 8.03%, 7.86%, 6.58%, 6.13%, respectively. The RS contribution rates of other trajectory variables were detailed in Fig. 7B.

Fig. 7C shows the detailed distribution of RS during each joint of each plane of kinematics and kinetics. There was revealing information contributing to the distribution of the time points variables between the before fatigue and after fatigue single-leg landing during the overground landing movement patterns.

A total of 169 relevant variables (RS greater than 0.7) were extracted as the notable highly relevant variable to explore its distribution trend (Fig. 7D). For the ankle kinematics, there was high RS in dorsiflexion-plantarflexion angle during the 15%–19% landing phase; in internal–external rotation angle during the 56%–60% landing phase. For the ankle kinetics, there was high RS in dorsiflexion-plantarflexion moment during the 2%–11%, 44%–50%, 56%–60%, 83%–85% landing phase; in inversion-eversion moment during the 50%–52% landing phase; in internal–external rotation moment during the 2%–5%, 18%–20% landing phase.

For the knee kinematics, there was high RS in flexion–extension angle during the 17%–24%, 72%–74%, 95%–99% landing phase; in abduction–adduction moment during the 29%, 67%–73% landing phase; in internal–external rotation angle during the 3%–5%, 17%–18%, 63%–69% landing phase. For the knee kinetics, there was high RS in flexion–extension moment during the 13%–30%, 41%, 72%–73% landing phase; in abduction–adduction moment during the 6%–10%, 28%–35% landing phase; in internal–external rotation moment during the 12%–14%, 28%–37%, 46%, 70%–73%, 77% landing phase.

For the hip kinematics, there was high RS in flexion–extension angle during the 9%–12%, 40%–45% landing phase; in abduction–adduction moment during the 62%–64% landing phase; in internal–external rotation angle during the 1%–3%, 12%–16% landing phase. For the hip kinetics, there was high RS in flexion–extension moment during the 11%–13%, 16%–19% landing phase; in abduction–adduction moment during the 4%–6%, 10%–13% landing phase. These high RS variables were also mainly concentrated in the early landing phase (red shaded area), which also suggested that the early stage landing phase was more important in landing pattern recognition.

## 4 Discussion

The primary purpose of this work was to investigate whether XML can help with landing pattern recognition and to what extent it can aid in the interpretation of prediction results. This work firstly compared the classification recognition performances of several classical classification models on two class landing tasks, and then constructed the XML model based on the neural network model with the best recognition performance combined with LRP to explain the model classification recognition results. Meanwhile, the RS results derived from LRP were evaluated from the statistical and clinical perspectives. Finally, we completed the validation of the following two questions: 1) which areas of input signals in the two class landing patterns are most relevant to the landing pattern recognition? 2) To what extent do these regions of input signals identified as most relevant for landing pattern recognition remain consistent with statistical evidence and clinical consensus assessment?

Before constructing the XML model, we first selected three classical and widely used classification algorithm models (SVM, ANN, CNN) for automated landing pattern classification tasks. From the current results, both three classification algorithm models achieved high recognizability in the nine classification tasks (Fig. 3, Table 1). In order to make a referenceable comparison of classification model performance to select the best classification model, we also compared it with the calculated ZRB besides comparing the differences between the models. This kind of theoretical accuracy resulting from assigning class labels based on the prior probability of the class represents the minimum at which the input signals between classes can be identified. There are potential risks in analyzing an unreliable classification model, and high identifiability is the basis for further interpretability analysis and the key to providing important information [27]. In other terms, only when the selected classification model can robustly identify the differences between the target classes, the subsequent construction of XML combined with LRP for objective interpretability analysis will be more trustworthy. Among the three classification algorithm models, the classification performance level of ANN for the input signal is particularly outstanding in comparison to the other two models, so the current work only provided the LRP analyzed with the ANN algorithm model. For the binary classification task, this approach undoubtedly reduces the potential risk to a large extent, and its better recognition performance can make the XML model extract more robust features in the input signal.

Which areas of input signals in the two class landing patterns are most relevant to the landing pattern recognition? From the classification performance, we found that based on the knee data as input signals the classification performance was better (Fig. 3B). This result suggests that the knee related landing patterns vary more between classes, that is, the contribution of the knee to the successful classification of the two classes of landing patterns may be greater. During landing, the lower limb undergoes a load transfer pattern from distal to proximal, in which the foot and ankle first bear the impact of the ground reaction force, followed by the knee and hip joints [1,2]. After the fatigue intervention, the degree of lower limb instability will be more severe during the landing phase. In response to this increased instability, the body subconsciously reduces knee and hip flexion during landing to maintain stability and avoid falling [44]. This process inevitably increases the impact on the lower limb musculoskeletal, thus changing the landing pattern [44]. As an intermediate joint connecting the distal (hip) and proximal (foot and ankle) joints, the knee joint plays a crucial role in the energy impact and transmission process of the lower limb movement chain [1]. Especially in single-leg landings, the energy transfer and impact force can only be absorbed by the lower limb muscle tissue in contact with the ground (landing leg). At this time, the knee joint plays a dominant role in absorbing impact and energy consumption of the whole lower limb [59]. Therefore, the movement trend of the knee joint is most pronounced under different control conditions, which results from the adaptive adjustment strategy produced by the connecting joint in response to the landing impact. This also results in the classification performance based on knee data as input signals are significantly better than that of the ankle and hip joints.

In terms of the classification performance, we found that the contribution of the sagittal plane to the successful classification of the two classes of landing patterns would be greater. Compared with the frontal and transversal planes, the range of motion of lower limb joints in the sagittal plane is significantly larger, allowing for greater movement. Also for landing tasks, the energy dissipated in the sagittal plane can reach 10–20 times the energy dissipated in other planes [1,59], so the main factor affecting the cushioning performance of the lower limb is the range of motion of the joints in the sagittal plane. Previous studies have shown that the most obvious change in landing pattern after fatigue intervention is a reduction in the degree of sagittal flexion of lower extremity joints, primarily in the knee joint [46,60]. The functional valgus collapse of the knee that traditionally leads to ACL injury is also directly related to the increased load impact caused by the stiffer landing mode [2]. Because at a small flexion angle of the knee joint, compared with the longitudinal force, the transverse pull force caused by the load impact in the vertical direction is the main force on the ACL [2]. In addition, the range of motion of each joint in the sagittal plane is much larger than that in the other planes, which is the main reason for the large variance in sagittal landing patterns between classes [44,46]. Therefore, it is convincing that there is a greater difference in sagittal landing patterns between classes in the three planes. However, are these features also consistent with the predicted interpretation of the results?

Prediction interpretation aims to explain the local behavior of the model, that is, to predict a given input signal and then explain which part of the input signal has the greatest impact on the prediction of the classifier [31]. The LRP used in this study is one of the prediction interpretations, which propagates something importantly relevant to the prediction from the output layer of the model to the input layer by backward propagation to determine the relative contribution of each input feature, and finally completes the relevant evidence identification of a specific prediction [32]. For landing pattern recognition, XML can highlight signal regions and characteristic signal shapes in the input signal that are associated with a particular landing pattern. So, to what extent do these regions of input signals identified as most relevant for landing pattern recognition remain consistent with statistical evidence? First, as we discussed above, of the three joints, the knee is probably the joint that contributes most to landing pattern recognition, and of the three planes, the sagittal plane is probably the plane that contributes most to landing pattern recognition. In statistical terms, the statistical differences between the two landing patterns are most significant in the sagittal plane and the knee joint. In conclusion, the RS results derived from the XML model are consistent with the statistical and clinical analysis in at least two respects.

From detailed results of RS distribution, we also found that the early landing phase contributed more to landing pattern recognition between classes. This is difficult for us to evaluate from a statistical point of view, because the statistical results can only be partitioned for a given region and cannot be quantified to a specific value at each time point. Therefore, this is why the current research proposed the combination of interpretable machine learning to help solve the problem of landing pattern recognition. The XML constructed by combining ANN and LRP is able to support the predicted explanatory output for each variable at different time points during the landing phase. This is because the early landing phase of single-leg landing is an extremely unstable state, while that phase is often accompanied by large impact loads, which leads to a sharp change in landing patterns between classes [8,46,54,61]. When the larger impact loads are piled up at the point of instability, the risk of lower limb injury naturally increases.

Combining the statistical and RS results during each joint of each plane, the RS in identifying the differences between the two class landing patterns generally agreed with the statistical results in the ankle and knee joints (Figs. 4 and 5). However, the statistical results and RS results in the frontal plane of the hip joint were inconsistent. This may be related to the fact that the uniqueness of landing patterns of individual subjects is also taken into account in the calculation of RS [11,32]. In contrast to statistical analyses where the input signal is based only on the average of individual subject characteristics, XML also takes into account the variability of the data for each test. This can lead to such discrepancies when the variability of the data is more disordered on a per-test basis. However, in most cases, when the XML model extracts general features of individual subjects for landing pattern recognition, its output RS can be convincing [62,63].

At this stage, an understanding of ML and AI decision making seems inevitable for the application of ML and AI in clinical injury screening, intervention, and treatment. The lack of transparency is a major problem faced in the application of ML and AI in the clinic, and there is an increasing demand for clinical experts to further interpret the prediction results of ML [28,62]. Here, we demonstrate the feasibility of interpreting machine learning predictions in landing pattern recognition by combining XML models constructed by LRP, which can not only satisfy the traditional decision problem between classes, but also largely solve the lack of transparency in landing pattern recognition.

Several potential limitations still should be considered in this work. First, only male subject data was collected to train the XML models in the current study, future studies should take into account data from female subjects to more comprehensively validate the effectiveness of the XML models for landing pattern recognition in clinical diagnosis. Secondly, we used only the datasets of before and after fatigue landings to validate the feasibility of the XML model in exploring the degree to which the biomechanics of each joint contribute to recognizing various landing patterns. Based on the consistency of the implementation scheme of this application on clinical patient datasets, we deduce that the proposed XML model is also applicable to the clinical situation of screening and diagnosis of injuries based on landing patterns. Future studies should also consider patient-based datasets to further demonstrate the utility of the proposed XML model for clinical diagnosis of landing patterns. In addition, the current work has visualized the recognition results in the form of color-coded waveform. For clinical application by non-specialists, future research should consider translating predictive interpretation results into an easily understandable text format.

## 5 Conclusion

In conclusion, the current work highlights the applicability of XML methods that can interpret the results of ML decisions for clinical landing analysis, and their great promise for future application and implementation. We explored the landing pattern recognition between classes, which provided a feasible framework for realizing the interpretability of ML decision results in clinical landing analysis, and provided methodological reference and a solid foundation for future clinical diagnosis and biomechanical analysis. Also, in order to facilitate future clinical applications, in addition to the color-coded waveform figures presented in the current study, translation of the predictive interpretation results into an easy-to-understand text format should be considered. This will enable the research method to be understood by more people, and also provide clinical experts with more in-depth and intuitive explanations when analyzing landing patterns.

## Funding statement

This study was sponsored by Research Academy of Medicine Combining Sports, Ningbo (No.2023001), the Project of NINGBO Leading Medical &Health Discipline (No.2022-F15, No.2022-F22), Zhejiang Key 10.13039/100006190Research and Development Program (Grant number: 2021C03130), Zhejiang Province Science Fund for Distinguished Young Scholars (Grant number: LR22A020002), Public Welfare Science and Technology Project of Ningbo, China (Grant number: 2021S134), Ningbo Key 10.13039/100006190Research and Development Program (Grant number: 20222ZDYF020016), 10.13039/100007834Ningbo Natural Science Foundation (Grant number: 2022J065, 2022J120) and K. C. Wong Magna Fund in 10.13039/501100004387Ningbo University. Datao Xu is being sponsored by the 10.13039/501100004543China Scholarship Council (10.13039/100014705CSC).

## Additional information

The Additional information file includes: (1) Supplementary Material Text 1 - Landing Pattern Data; (2) Supplementary Material Text 2 - Classification Methods; (3) SI References.

## Data availability statement

All data relevant to the current study are included in the article and supplementary information, further inquiries can be directed to the corresponding author.

## CRediT authorship contribution statement

**Datao Xu:** Writing – original draft, Visualization, Software, Methodology, Investigation, Formal analysis, Conceptualization. **Huiyu Zhou:** Writing – review & editing, Visualization, Supervision, Resources, Methodology, Investigation, Conceptualization. **Wenjing Quan:** Writing – review & editing, Methodology, Investigation, Conceptualization. **Ukadike Chris Ugbolue:** Writing – review & editing, Resources, Investigation. **Fekete Gusztav:** Writing – review & editing, Supervision, Resources, Methodology, Conceptualization. **Yaodong Gu:** Writing – review & editing, Visualization, Supervision, Software, Methodology, Investigation, Funding acquisition, Conceptualization.

## Declaration of competing interest

The authors declare that they have no known competing financial interests or personal relationships that could have appeared to influence the work reported in this paper.
