# Supplementary material for: A new method applied for explaining the landing patterns: Interpretability analysis of machine learning
Source: Heliyon. 2024 Feb 9;10(4):e26052. doi: 10.1016/j.heliyon.2024.e26052 (PMC10869904; doi:10.1016/j.heliyon.2024.e26052)
Supplement: Multimedia component 1 [file mmc1.pdf]

# 1. Supplementary Material Text 1 - Landing Pattern Data

## 1.1 Subjects

A total of 56 healthy male subjects (age: 22.56±5.13 years; body mass: 82.62±13.38 kg; height: 1.85±0.11 m) were recruited for this study. To be eligible, participants had to have no history of severe lower-extremity surgery or any other injury variables that would interfere with the study within the previous six months. The experiment's goals, requirements, and procedures were explained to the participants. Ningbo University's Ethics Committee has accepted the study protocol (Approval Number: RAGH20210120), and all subjects supplied and signed written informed permission.

## 1.2 Experimental Protocol and Procedures

The Vicon motion capture device with 8 infrared cameras (Vicon Metrics Ltd., United Kingdom) was used to obtain three-dimensional lower limb joint kinematic data at a 200 Hz sampling frequency. Meanwhile, the ground reaction force (GRF) data were obtained synchronously using a 1000 Hz AMTI force plate installed in the ground (AMTI, Watertown, United States). The experiment was divided into three parts: 1) landing biomechanical test before fatigue intervention; 2) fatigue intervention; 3) landing biomechanical test after fatigue intervention. The process of biomechanical tests before and after fatigue intervention was the same. All subjects were required to wear tights, leggings, and uniform shoes, and warmed up for 10 minutes by running at

their own pace in the laboratory, then they acquainted themselves with the experimental procedure.

This study performed musculoskeletal modelling of the lower limbs based on the pipeline that had been constructed from previous models [1, 2]. The dominant leg was chosen as the analytical limb in this study, and the reflective markers (12.5 mm diameter standard) were affixed to the pelvic and lower limb according to procedures outlined in the previous study (**Figure S1A**). The placement of reflective markers in all subjects was performed by the same experimenter and checked by another experimenter. After the reflective markers were affixed, subjects were asked to stand on the force plate in a standard anatomical posture with their feet open, shoulder width apart, arms open to 45° oblique downward, and they were visually ahead and kept motionless until the experimenter completed static data collection.

The experiment was divided into three parts: 1) Landing biomechanical test of before fatigue intervention; 2) Fatigue intervention; 3) Landing biomechanical test of after fatigue intervention (**Figure S1B**). The drop landing was selected as the landing test maneuver, and the whole test process is outlined in **Figure S1C**. As a general landing test maneuver, drop landing maximizes the revivification of landing properties, and has been used in a large number of studies [3, 4]. A 40 cm high jump platform was placed directly in front of the force plate, and the subject stood on the jump platform with his hands on his hips. After hearing the "begin" signal from the experimenter, the subject moves the dominant leg forward, and leans forward to fall vertically from the jump platform at no initial speed. Subjects were instructed to land with their dominant

leg as close to the center of the force plate as possible, and then land on one leg for support and balance. A successful experiment was defined as the subject's ability to balance on the dominant leg for 3 seconds without any tendency to fall. 5 successful landing trial datasets were collected for each subject both before and after the fatigue intervention landing test. The subjects rested for at least 30 seconds between each session before the fatigue intervention landing test, and their heart rates were monitored using a wireless Polar table to ensure their heart rates had recovered before the next testing.

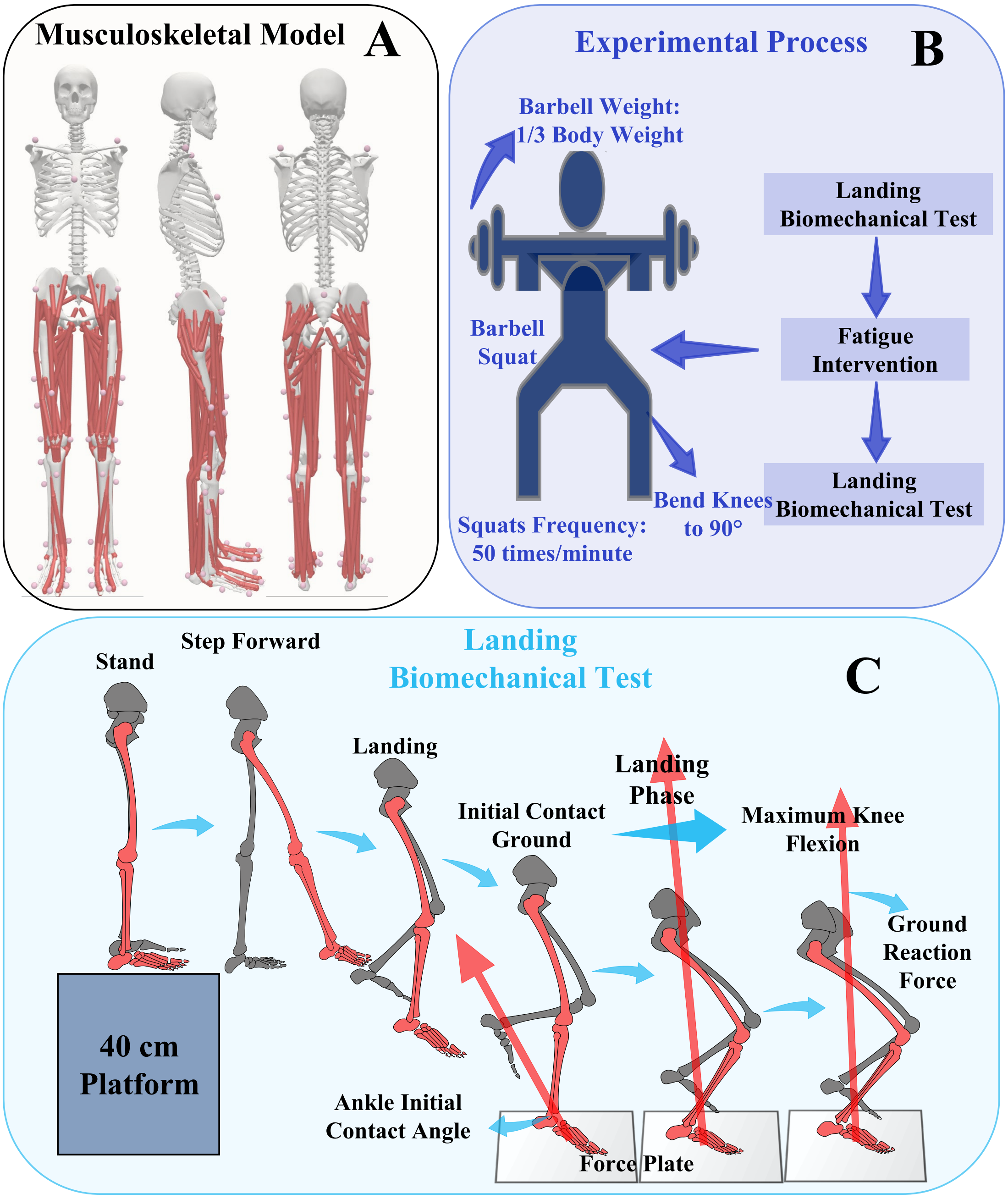


**Figure S1.** Overview of the musculoskeletal model and whole study experimental procedure. **Figure S1A** Illustration of the position of the reflective marking points about the constructed musculoskeletal model. **Figure S1B** Illustration of the whole experimental process, which includes three main steps: 1) Landing biomechanics test of before fatigue intervention; 2) Fatigue intervention; 3) Landing biomechanics test of after fatigue intervention. **Figure S1C** Illustration of the process of landing

biomechanics test. The landing phase was from initial contact with the ground to maximum knee flexion.

Fatigue intervention was performed immediately after the completion of the before fatigue landing biomechanical test. The current study adopts the continuous close chain action in the whole process to better maintain and control the state of lower extremity muscle fatigue. Specifically, subjects loaded the barbell with their knees bent to 90° to squat, the weight of the barbell was set to 1/3 of the subject's body weight, and the frequency of squatting was set to 50 times/min [5, 6]. During the fatigue intervention, two experimenters assisted the subjects to carry out the fatigue intervention. The experimenter first selected the barbell plate according to the body weight of the subjects. Then, the experimenter set the metronome at a frequency of 50 times/min, and the subjects performed weight-bearing squats according to the rhythm of the metronome. Throughout the fatigue intervention, the subjects were protected and verbal encouragement was given as motivation.

Subject were considered fatigued based on the following criteria: 1) Subjects cannot complete 2 effective squats or cannot keep up with 4 consecutive squats; 2) According to the session rating of perceived exertion (PRE) to determine the subjects' subjective fatigue. PRE is divided into 6 to 20 levels, level 6 is defined as quiet, and level 20 is defined as very tired [7]. In this study, fatigue was defined as an RPE level above 17 [8]; 3) Heart rate reaches 85% of maximum heart rate, and maximum heart rate was defined as the 220 minus subject’s age [9]. When the subject met any one of

the three criteria, the subject was considered to be fatigued and was permitted to begin the landing test.

Landing tests after fatigue intervention was performed as soon as the subject was determined to be maximally fatigued. The landing test requirements were the same as those before fatigue, but there was no rest time between each session during after fatigue landing test. Meanwhile, the subjects were asked to perform the squat jump three times in a row between each test to maintain the fatigue effect [10]. The time to complete the landing movement and the immediate heart rate were recorded throughout the test, and the results are shown in **Table S1**.

**Table S1.** The physical state of athletes during the experiment.

| | Complete time (s) | Heart Rate (times/min) | PRE-Level |
|---|---|---|---|
| Landing Tests Before Fatigue Intervention | 287±79.1 | 82.3±6.9 | 8.0±0.5 |
| Fatigue Intervention | 123.6±23.5 | 165.6±10.1 | 17.0±1.5 |
| Landing Tests After Fatigue Intervention | 169.6±68.4 | 172.3±9.8 | 17.5±1.1 |

## 1.3 Data Analysis and Processing

Vertical GRF greater than 10N was used to establish the initial contact force point [11, 12]. The landing phase was defined as from the initial contact force point to the

maximum knee flexion [11, 13]. The data was initially processed in Vicon Nexus software: 1) Name the captured reflective markers; 2) fix the missing reflective markers in this stage; 3) delete the redundant and wrong reflective markers. Data were collected for a total of 5 s, including 2 s before and 3 s following the initial contact with the ground [4]. Then, the exported C3D files from Vicon Nexus were imported into Visual 3D 6.7.3 (C-Motion Inc., Germantown, US) software for further modeling process [14]. In this step, joint kinematics (joint angle) and joint kinetics (joint moment) are calculated by using the built-in inverse kinematics and inverse dynamics algorithms in Visual3D. Based on Winter's study of the selected filter frequency [15], the most appropriate signal-to-noise ratio was determined by carrying out the residual analysis of data in subsets. Based on this, fourth-order zero-phase lag Butterworth low-pass filters with frequencies of 10 and 20 Hz were used to filter the kinematic and kinetic data. All the kinematics and kinetics data of each joint (ankle, knee, hip) of each plane (sagittal, frontal, transversal) were then imported into MATLAB R2022a (Visual R2022a, MathWorks, United States) and expanded into 101 data point curves by a custom MATLAB script. Finally, the data matrices were obtained as follow:

$$M_{before\ fatigue} = 280(56_{subjects} \times 5_{trials}) \times 202(101_{kinematics} + 101_{kinetics}) \times 9(3_{joint} \times 3_{plane});$$

$$M_{after\ fatigue} = 280(56_{subjects} \times 5_{trials}) \times 202(101_{kinematics} + 101_{kinetics}) \times 9(3_{joint} \times 3_{plane}).$$

# 2. Supplementary Material Text 2- Classification Methods

The SVM has many unique advantages in solving nonlinear, small-sample, high-dimensional data pattern recognition problems. Given training sample set $D = \{(x_1, y_1), (x_2, y_2), \dots, (x_m, y_m)\}, y_i \in \{-1, +1\}$, the essence of classification is to find a partition hyperplane in the sample space to separate samples of different categories. For the SVM, the model corresponding to the hyperplane partition in the feature space can be expressed as $f(x) = \omega^T \phi(x) + b$. Where the $\omega = (\omega_1; \omega_2; \dots; \omega_d)$ is the normal vector that determines the direction of the hyperplane; the $b$ is the displacement term, which determines the distance between the hyperplane and the origin; the $\phi(x)$ represents the eigenvectors after the $x$ map. By setting the kernel function $\kappa(x_i, y_i) = \phi(x_i)^T \phi(x_j)$, the support vector expansion can be obtained:

$$f(x) = \omega^T \phi(x) + b = \sum_{i=1}^{m} \alpha_i y_i \phi(x_i)^T \phi(x) + b = \sum_{i=1}^{m} \alpha_i y_i \kappa(x, x_i) + b \quad (1)$$

In this study, the linear kernel functions ($\kappa(x, x_i) = {x_i}^T x_j$) were used to turn the input feature's data into a higher-dimensional space. At the same time, the soft margin idea was used to cope with the possibility of misclassifications. The soft margin should make the samples that do not meet the constraints as small as possible while maximizing the margin, so that the optimization objective can be expressed as: $min_{\omega,b,\xi_i} \frac{1}{2} ||\omega||^2 + C \sum_{i=1}^{m} \xi_i$. Where the $\xi_i$ is the slack variables, and the $C$ ($C = 1$) is a regularization constant [16, 17].

In neural networks, "weight sharing" is a strategy for saving training overhead by having a group of neurons use the same connection weights, and this strategy plays an

important role in CNN [18, 19]. CNN is a feed-forward neural network, mainly inspired by natural biological visual cognitive mechanisms, which consists of the convolutional layer, pooling layer, and fully connected layer [18]. The current study processes the input signal by composing three successive convolutional and pooling layers, and then implements a mapping to the output target in a fully connected layer. Each convolutional layer contains multiple feature maps, each of which is a 'plane' of multiple neurons that extract a feature of the input through a convolutional filter. The Rectified Linear Unit was used as the activation function, the top of the fully connected layers activated using a SoftMax output, with the filter size and stride configuration of 1-4, and the number of filters in the convolutional layer was set to 32.

ANN are extensive parallel networks comprised of adaptable basic units whose organization can be used to replicate the interactions of organic nervous systems with real-world objects [20, 21]. In this study, an ANN model with ten hidden layers was created under the condition of repeated model training and adjustment following the actual data, as the application of the ANN model in the current work was primarily skewed to increase the model's accuracy [22, 23]. The layers of the neural network are completely connected, that is, any neuron of the n-th layer must be connected to any neuron of the n+1 layer. Through forward propagation, the linear relation function and activation function continuously calculate the new values in a cycle from layer to layer, and finally obtain the model results. The linear relationship function of the model constructed in this study is: $z = \sum_{i=1}^{m} w_i x_i + b$, where the $w_i$ is the connection weight of the $i$-th neuron, the $x_i$ is the input from the $i$-th neuron. Take the input $x$

and run it linearly to get $z$, and then the Sigmoid ($S = \frac{1}{1+e^{-x}}$) type activation function is used to get $a$ [22].

Take the input $x$ and run it linearly to get $z$, and then the hyperbolic tangent activation function is used to get $a$. Therefore, for the output of the 2-th layer, the $a_1^2$, $a_2^2$, $a_3^2$ can be obtained:

$$a_1^2 = \sigma(z_1^2) = \sigma(w_{11}^2 x_1 + w_{12}^2 x_2 + w_{13}^2 x_3 + b_1^2)$$

$$a_2^2 = \sigma(z_2^2) = \sigma(w_{21}^2 x_1 + w_{22}^2 x_2 + w_{23}^2 x_3 + b_2^2)$$

$$a_3^2 = \sigma(z_3^2) = \sigma(w_{31}^2 x_1 + w_{32}^2 x_2 + w_{33}^2 x_3 + b_3^2)$$

For the output of the 3-th layer:

$$a_1^3 = \sigma(z_1^3) = \sigma(w_{11}^3 a_1^2 + w_{12}^3 a_2^2 + w_{13}^3 a_3^2 + b_1^3)$$

Finally, the neural network output $a_i^{l+1}$ of the $i$-th neuron at layer $l+1$ is:

$$f(x) = a_i^{l+1} = \sigma\left(z_i^{l+1}\right) = \sigma\left(\sum_i a_i^{(l)} w_{ij}^{(l,l+1)} + b_j^{(l+1)}\right) \quad (2)$$

Where $i$ is a neuron at the layer $l+1$, the $\sum_i$ ... runs over all lower layer neurons that are connected to neuron $j$, the $w_{ij}^{(l,l+1)}$, $b_j^{(l+1)}$ are specific parameters to pairs of adjacent neurons. The node of the input layer was determined according to the number of input features, the node of the hidden layer was determined according to the group number of input data, and the node of the output layer was determined based on the number of classes, and batch size was set as 25, the max epoch was set to 3000 [22].

## 3. References

[1] S.L. Delp, F.C. Anderson, A.S. Arnold, P. Loan, A. Habib, C.T. John, E. Guendelman, D.G. Thelen, OpenSim: open-source software to create and analyze dynamic simulations of movement, IEEE. T. Bio-Med. Eng. 54 (2007) 1940-1950. https://doi.org/10.1109/TBME.2007.901024

[2] A. Sikidar, M. Marieswaran, D. Kalyanasundaram, Estimation of forces on anterior cruciate ligament in dynamic activities, Biomech. Model. Mechan. 20 (2021) 1533-1546. https://doi.org/10.1007/s10237-021-01461-5

[3] D. Xu, Z. Lu, S. Shen, G. Fekete, U.C. Ugbolue, Y. Gu, The Differences in lower extremity joints energy dissipation strategy during landing between athletes with symptomatic patellar tendinopathy (PT) and without patellar tendinopathy (UPT), Mol. Cell. Biomech. 18 (2021). https://doi.org/10.32604/mcb.2021.015453

[4] D. Xu, H. Zhou, J.S. Baker, B. István, Y. Gu, An investigation of differences in lower extremity biomechanics during single-leg landing from height using bionic shoes and normal shoes, Front. Bioeng. Biotech. 9 (2021) 711. https://doi.org/10.3389/fbioe.2021.679123

[5] R. Haddas, C.R. James, T.L. Hooper, Lower extremity fatigue, sex, and landing performance in a population with recurrent low back pain, J. Athl. Training. 50 (2015) 378-384. https://doi.org/10.4085/1062-6050-49.3.61

[6] D.A. Padua, B.L. Arnold, D.H. Perrin, B.M. Gansneder, C.R. Carcia, K.P. Granata, Fatigue, vertical leg stiffness, and stiffness control strategies in males and females, J. Athl. Training. 41 (2006) 294. https://doi.org/10.1007/s10237-021-01461-5

[7] J.P. Buckley, G.A. Borg, Borg's scales in strength training; from theory to practice in young and older adults, Appl. Physiol. Nutr. Me. 36 (2011) 682-692. https://doi.org/10.1139/h11-078

[8] S. Steib, A. Zech, C. Hentschke, K. Pfeifer, Fatigue-induced alterations of static and dynamic postural control in athletes with a history of ankle sprain, J. Athl. Training. 48 (2013) 203-208. https://doi.org/10.4085/1062-6050-48.1.08

[9] N. Cortes, D. Quammen, S. Lucci, E. Greska, J. Onate, A functional agility short-term fatigue protocol changes lower extremity mechanics, J. Sport. Sci. 30 (2012) 797-805. https://doi.org/10.1080/02640414.2012.671528

[10] D. Quammen, N. Cortes, B.L. Van Lunen, S. Lucci, S.I. Ringleb, J. Onate, Two different fatigue protocols and lower extremity motion patterns during a stop-jump task, J. Athl. Training. 47 (2012) 32-41. https://doi.org/10.4085/1062-6050-47.1.32

[11] D. Xu, X. Jiang, X. Cen, J.S. Baker, Y. Gu, Single-leg landings following a volleyball spike may increase the risk of anterior cruciate ligament injury more than landing on both-legs, Appl. Sci. 11 (2020) 130. https://doi.org/10.3390/app11010130

[12] D. Xu, J. Lu, J.S. Baker, G. Fekete, Y. Gu, Temporal kinematic and kinetics differences throughout different landing ways following volleyball spike shots, P. I. Mech. Eng. P-J. Spo. 236 (2021) 1-8. https://doi.org/10.1177/17543371211009485

[13] D. Xu, X. Cen, M. Wang, M. Rong, B. István, J.S. Baker, Y. Gu, Temporal

kinematic differences between forward and backward jump-landing, Int. J. Env. Res. Pub. He. 17 (2020) 6669. https://doi.org/10.3390/ijerph17186669

[14] H. Zhou, D. Xu, W. Quan, M. Liang, U.C. Ugbolue, J.S. Baker, Y. Gu, A pilot study of muscle force between normal shoes and bionic shoes during men walking and running stance phase using opensim, Actuators, 10 (2021) 274. https://doi.org/10.3390/act10100274

[15] D.A. Winter, Biomechanics and motor control of human movement, John Wiley & Sons (2009).

[16] C. Cortes, V. Vapnik, Support-vector networks, Mach. Learn.20 (1995) 273-297.

[17] S. Suthaharan, Machine learning models and algorithms for big data classification, Integr. Ser. Inf. Syst. 36 (2016) 1-12.

[18] Y. LeCun, Y. Bengio, Convolutional networks for images, speech, and time series, The handbook of brain theory and neural networks, 3361 (1995) 1-10.

[19] Y. LeCun, L. Bottou, Y. Bengio, P. Haffner, Gradient-based learning applied to document recognition, P. IEEE. 86 (1998) 2278-2324. https://doi.org/10.1109/5.726791

[20] O.I. Abiodun, A. Jantan, A.E. Omolara, K.V. Dada, N.A. Mohamed, H. Arshad, State-of-the-art in artificial neural network applications: A survey, Heliyon. 4 (2018) e00938. https://doi.org/10.1016/j.heliyon.2018.e00938

[21] T. Kohonen, An introduction to neural computing, Neural. Networks. 1 (1988) 3-16. https://doi.org/10.1016/0893-6080(88)90020-2

[22] D. Xu, W. Quan, H. Zhou, D. Sun, J.S. Baker, Y. Gu, Explaining the differences of

gait patterns between high and low-mileage runners with machine learning, Sci. Rep. 12 (2022) 1-12. https://doi.org/10.1038/s41598-022-07054-1

[23] W. Liu, Z. Wang, X. Liu, N. Zeng, Y. Liu, F.E. Alsaadi, A survey of deep neural network architectures and their applications, Neurocomputing 234 (2017) 11-26. https://doi.org/10.1016/j.neucom.2016.12.038
